# Mast Cell Protease 7 Promotes Angiogenesis by Degradation of Integrin Subunits

**DOI:** 10.3390/cells8040349

**Published:** 2019-04-12

**Authors:** Devandir A. de Souza Junior, Carolina Santana, Gabriel V. Vieira, Constance Oliver, Maria Celia Jamur

**Affiliations:** Department of Cell and Molecular Biology and Pathogenic Bioagents, Ribeirão Preto Medical School, University of São Paulo, Ribeirão Preto 14.049-900, Brazil; dasjunior@hotmail.com (D.A.d.S.J.); carousantana@gmail.com (C.S.); gabrielviliod@gmail.com (G.V.V.); coliver@fmrp.usp.br (C.O.)

**Keywords:** mast cell, angiogenesis, proteases, integrin, ubiquitin/proteasome, DIVAA™

## Abstract

Previous studies from our laboratory have shown that during angiogenesis in vitro, rmMCP-7 (recombinant mouse mast cell protease-7) stimulates endothelial cell spreading and induces their penetration into the matrix. The ability of rmMCP-7 to induce angiogenesis in vivo was assessed in the present study using a directed in vivo angiogenesis assay (DIVAA™). Vessel invasion of the angioreactor was observed in the presence of rmMCP-7 but was not seen in the control. Since integrins are involved in endothelial cell migration, the relationship between rmMCP-7 and integrins during angiogenesis was investigated. Incubation with rmMCP-7 resulted in a reduction in the levels of integrin subunits αv and β1 on SVEC4-10 endothelial cells during angiogenesis in vitro. Furthermore, the degradation of integrin subunits occurs both through the direct action of rmMCP-7 and indirectly via the ubiquitin/proteasome system. Even in the presence of a proteasome inhibitor, incubation of endothelial cells with rmMCP-7 induced cell migration and tube formation as well as the beginning of loop formation. These data indicate that the direct degradation of the integrin subunits by rmMCP-7 is sufficient to initiate angiogenesis. The results demonstrate, for the first time, that mMCP-7 acts in angiogenesis through integrin degradation.

## 1. Introduction

Angiogenesis is a dynamic process that consists of the formation of new blood vessels from pre-existing ones which begins in the embryo and continues throughout life [1,2]. Angiogenesis is regulated by the balance between different angiogenic factors and endothelial cell interactions with the components of the extracellular matrix, adhesion molecules, and mechanical forces [1,2,3,4,5]. Angiogenesis consists of three basic stages: the first, selection of some endothelial cells inside the capillary to begin angiogenic expansion. These cells react to the angiogenic factor VEGF-A (vascular endothelial growth factor) and initiate invasion and migration. The second stage (migration, endothelial cell proliferation, and tube formation) is mediated by the interaction of VEGF-A and VEGFR-2. Maturation of newly formed blood vessels occurs in the third stage. Proliferation and migration of endothelial cells is inhibited, and the newly formed vessels fuse with other vessels, both new vessels and pre-existing vessels. This fusion stabilizes the newly formed vessels and forms a functional vascular network [6,7,8,9]. Alterations in this process which involve the relationship between the endothelial cells and their microenvironment may compromise the development and function of blood vessels [10]. Thus, angiogenic factors and regulatory components of cell spreading, such as migration and adhesion, have been the target of several studies [11,12,13,14,15].

Recent studies have shown that other cells types, such as macrophages, neutrophils, eosinophils, mast cells, and dendritic cells, have an important role in regulating the formation and maintenance of blood vessels during angiogenesis [11,16,17,18,19]. Signals produced within the tissue microenvironment appear to stimulate many of the pro-angiogenic functions of these cells on endothelial cells [20]. However, regulatory mechanisms involved in cell spreading and endothelial branching during angiogenesis, as well as, the cell types and/or proteins involved in these processes remain unclear.

Mast cells are connective tissue cells which are involved in host defense and participate in allergic and inflammatory reactions [21,22,23,24,25]. The ability of mast cells to produce and release a wide range of chemical mediators is essential for their function in different physiological and pathological processes [21,26,27,28]. The exact repertoire of expressed mast cell proteases varies from species to species, and these differences are used to phenotypically classify mast cells. In humans, the four homologous tryptases (α and βI-III) that have been cloned reside at a complex on chromosome [29,30,31]. In mouse, only two tetrameric tryptases (designated mouse MC protease (mMCP) 6 and mMCP-7) have been identified so far. Their genes are located on mouse chromosome 17A3 [32,33,34]. The mouse and human TPSB2 and TPSAB1 genes encode tetramer-forming tryptases stored in the secretory granules of mast cells. The homolog of mMCP-7 in humans is tryptase alpha/beta2 [29,35,36,37]. Despite the chromosomal clustering of their genes, these mouse tryptases are differentially regulated in vivo [38,39,40,41] and in vitro [42,43] at the levels of gene transcription [44] and mRNA stability [42].

Mast cells often accumulate near blood vessels and it is widely recognized that mast cells are associated with newly formed microvasculature [45,46,47]. Various lines of evidence suggested that secreting mast cells can induce and enhance angiogenesis via multiple, and partially interacting pathways [48,49,50,51]. Several of these studies have shown that some mediators released by mast cells such as heparin, histamine, TNF-α, bFGF, stimulate endothelial cell proliferation, thereby influencing angiogenesis [52,53,54,55,56,57].

Previous investigations from our laboratory have shown that mast cells are involved in the induction of angiogenesis in the early stages of tumor development and in modulating blood vessel growth at later stages of tumor progression. Although mast cell numbers increased during the early stages of tumor progression, chymase (mMCP-5), tryptase (mMCP-6 and 7), and carboxypeptidase A activity increased at all stages [55]. Further studies suggested that the subtypes of proteases released by mast cells could influence endothelial cells during in vivo neo-vascularization. Both rmMCP-6 and rmMCP-7 were able to accelerate tube formation and matrix invasion by SVEC4-10 endothelial cells. rmMCP-6 and rmMCP-7 were also able to induce the differential release of angiogenic factors from these cells [58]. Furthermore, rmMCP-7 was more efficient in stimulating tube formation and release of angiogenic factors than rmMCP-6 [58]. Additional investigations revealed that mast cells have both direct and indirect effects on angiogenesis. When endothelial cells were co-cultured with mast cells, angiogenesis was stimulated. Furthermore, there was direct intercellular communication via gap junctions between the mast cells and the endothelial cells. Moreover, the presence of either mast cells or conditioned medium from the co-cultures stimulated endothelial cells to release angiogenic factors [59].

Our previous study [58] demonstrated that in the presence of rmMCP-7, endothelial cell spreading and migration are stimulated which increases tube and loop formation, as well as increasing matrix invasion. The present study was undertaken to further elucidate the role of rmMCP-7 in in vitro and in vivo angiogenesis. The results demonstrate that rmMCP-7 directly degrades integrin subunits, thus facilitating migration, invasion, and/or adhesion of endothelial cells. These processes are essential for angiogenesis. Knowledge of the interaction between mast cells and their specific proteases on endothelial cells during angiogenesis contributes to our understanding of neovascularization.

## 2. Materials and Methods

### 2.1. Cells

The murine endothelial cell line SVEC4-10 (CRL-2181) was purchased from the American Type Culture Collection (ATCC; Manassas, VA, USA). The cell line was maintained in Dulbecco’s Modified Eagle’s Medium (DMEM) plus 10% heat inactivated fetal bovine serum (FBS). Cells were cultured at 37 °C in a humidified environment containing 5% CO_2_ in air. All reagents used for cell culture were purchased from Thermo Fisher Scientific (Invitrogen, Thermo Fisher Scientific, Carlsbad, CA, USA).

### 2.2. Cells Invasion Assay

The cell invasion assay was modified from Bayless et al. [60]. Briefly, a Secure-Seal^®^ Hybridization Chamber Gasket (Thermo Fisher Scientific) was adhered to the well of a glass Lab-Tek^®^ Chamber Slide™ (Electron Microscopy Sciences; Hatfield, PA, USA). Subsequently 20 μL of Geltrex^®^ was added through one port on the Secure-Seal^®^ coverslip. The slides were incubated for 30 min at 37 °C for gel solidification. 5 × 10^4^ cells were then added through the other port on the Secure-Seal^®^ chamber and the samples were incubated for 5 h in the presence or absence of rmMCP-7. After incubation the samples were fixed with 2% paraformaldehyde for 20 min and washed in PBS. The slides were analyzed using a Leica DMI600B9 microscope (Leica Systems, Wetzlar, Germany) and the images were acquired using a Leica DFC365FX (Leica) digital camera. The distance traveled by the cells was analyzed using the ImageJ program [61].

### 2.3. Directed In Vivo Angiogenesis Assay^TM^ (DIVAA^TM^)

Male nude mice, 4 weeks of age, obtained from the Center for the Breeding of Special Mice, Ribeirão Preto Medical School, were used for these experiments. Mice were housed under approved conditions in the Animal Research Facilities of the Ribeirão Preto Medical School and the research was conducted according to the guidelines of the Ribeirão Preto Medical School (protocol number 017/2019). For these experiments a commercially available quantitative angiogenesis kit (DIVAA^TM^) (Trevigen Inc., Gaithersburg, MD, USA) was used and the assays were done according to the manufacturer’s protocol. Briefly, angioreactors were filled with Geltrex^®^ only (negative control), Geltrex^®^ plus 37.5 ng VEGF and 12.5 ng FGF-2 (fibroblast growth factor-2) (positive control) or Geltrex^®^ plus 20ng of rmMCP-7. The angioreactors were incubated at 37 °C for 1 h to allow for gel solidification. Each group consisted of three mice and an angioreactor was implanted subcutaneously into the left and right dorsal flank of each mouse. Thirteen days after implantation, the mice were euthanized in a CO_2_ chamber at a flow rate of 20–30% CO_2_ per min according to Ribeirão Preto Medical School, Ribeirão Preto, Brazil guidelines and the angioreactors removed, photographed, and processed for analysis. Each angioreactor was flushed with 300 μL of CellSperse^TM^, and the content transferred to a microtube and incubated at 37 °C for 2 h. After incubation the samples were washed by centrifugation with DIVAA^TM^ Wash Buffer. The pellet was resuspended in 200 μL of DIVAA^TM^ FITC-Lectin and incubated at 4 °C overnight. After incubation, the samples were washed with DIVAA^TM^ Wash Buffer for fluorometric analysis. Fluorescence intensity was determined in an Elisa Power Wave X Plate Reader (Bio-Tek Instruments, Inc., Winooski, VT, USA) and expressed as mean relative fluorescence (excitation 485 nm, emission 510 nm).

### 2.4. In Vitro Angiogenesis: Tube Formation Assay

The tube formation assay measures the ability of endothelial cells to form tubes (capillary-like structures). In order to perform this assay, 10 μL of Geltrex^®^ (Invitrogen, Thermo Fisher Scientific) was added to the wells of μ-slides Angiogenesis^®^ (IBIDI, Martinsried, Germany) and allowed to solidify at 37 °C for 30 min. After the gel solidified, SVEC4-10 cells (1 × 10^4^) were added in 50 μL of DMEM supplemented with 10% FBS. The cells were incubated at 37 °C in a humidified atmosphere containing 5% CO_2_ in air for 5 h and then analyzed. The following parameters were used for quantification [62,63,64,65]:

Tubes were considered to be tubular structures that extend from one branching point to another branching point or to a loose end. Loops are enclosed (or almost enclosed) areas inside the tubes that fulfill roundness conditions.

The images were acquired using a Nikon Eclipse TE2000-U microscope (Nikon USA, Melville, NY, USA) equipped with a 10× objective. The measurements are expressed in pixels (800 × 600) where 1 pixel equals 0.069 mm^2^. WimTube (Wimasis Image Analysis, Munich, Germany; www.wimasis.com/en/products/13/WimTube) was used to analyze and quantify tube formation.

### 2.5. Expression of Integrin Subunits

Endothelial cells were cultured in the presence or absence of 20 ng rmMCP-7 (R and D Systems Inc., Minneapolis, MN, USA). The cells were incubated at 37 °C in a humidified atmosphere containing 5% CO_2_ in air for 5 h and then analyzed. After incubation the cells were lysed using RIPA lysis buffer (Invitrogen, Thermo Fisher Scientific) with 15µg/mL protease inhibitor cocktail (Millipore Sigma, St. Louis, MO, USA) for 20 min. Subsequently the lysate was centrifuged for 25 min at 14,000× *g* and the protein concentration of the supernatant was determined using the BCA Protein Assay Kit (Pierce, Thermo Fisher Scientific, Rockford, IL, USA). 20 µg of lysate were boiled for 5 min in SDS sample buffer (50 mM Tris-HCl pH 6.8, 12.5% glycerol, 1% sodium dodecylsulfate, 0.01% bromophenol blue), and applied to 10% polyacrylamide gels. Proteins were then separated electrophoretically and transferred to Hybond membranes (GE Healthcare Life Science, Pittsburgh, PA, USA). Membranes were blocked overnight in TBS (0.05 M Tris–HCl, 0.15 M NaCl, pH 7.5, and 0.05% Tween 20) containing 5% nonfat dry milk at 4 ℃. Membranes were then incubated with rabbit anti-integrin subunit (αv, α2, β1) or rat anti-integrin subunit α6 IgG ( all from Millipore Sigma, Burlington, MA, USA) at 1:200 for 1 h at RT, washed in TBS/Tween and incubated with goat anti-rabbit IgG conjugated to HRP or goat anti-rat IgG conjugated to HRP (1:20,000) (Jackson ImmunoResearch, West Grove, PA, USA) for 30 min at RT, washed and developed using chemiluminescence (ECL-GE Healthcare, Piscataway, NJ, USA). α-actin (Millipore Sigma) was used as a loading control. Images were obtained by exposing membranes to Bio-Rad Chemidoc (Bio-Rad, Hercules, CA, USA), and the bands analyzed with ImageLab software (Bio-Rad). The optical densities of the bands were determined using Adobe Photoshop (Adobe Systems, San Jose, CA, USA).

### 2.6. Integrin Degradation Assay

To analyze the direct degradation of integrin subunits αv and β1 (R and D Systems Inc.), the integrin subunits were incubated at 37 °C in the presence (1 ng rmMCP-7 to 10 ng integrin subunit) or in the absence of protease. After different times of incubation (15 min, 30 min, 1 h, 2 h 3 h, 4 h, and 5 h) the integrin subunits were analyzed by immunoblotting as previously described in Section 2.5.

### 2.7. Proteasome Inhibition

Proteasome inhibition was induced by incubation with MG132 (Calbiochem, San Diego, CA, USA). MG132 was dissolved in dimetylsufoxide (DMSO, Sigma-Aldrich, St. Louis, MO, USA) at 10 mM as a stock solution. The optimal concentration of MG132 for blocking tube formation was determined by a dose-response curve. SVEC4-10 cells were incubated with rmMCP-7 protease in the presence or absence of 25 µM MG132 for 5 h, as previously described in Section 2.4. As a control, cells were incubated in the presence or absence of MG132, without protease. After incubation the samples were analyzed by microscopy as described in Section 2.2.

### 2.8. Immunoprecitions Assay Using Anti-Ubiquitin

After the in vitro angiogenesis assay, the SVEC4-10 endothelial cells were lysed and the immunoprecipitation was performed using Kit-Dynabeads® Protein G (Invitrogen, Thermo Fisher Scientific) following the manufacturer’s guidelines. The magnetic beads were conjugated to anti- mouse ubiquitin FK2 (Millipore Sigma). Protein lysates were incubated with the Dynabeads^®^-Ab complex for 10 min at room temperature. After incubation the Dynabeads^®^-Ab-Ag complex was washed and the target antigen eluted. Immunoprecipitated proteins were analyzed by Western blotting with specific antibodies to detect the subunits of integrins (αv and β1). Protein values of the subunits were quantified using Adobe Photoshop.

### 2.9. Statistical Analysis

The data is expressed as mean ± SD from at least three independent experiments. In order to compare data, Student’s t-test was used. *p*-values of ≤0.05 were considered significant. Statistical analysis was performed with Graphpad Prism 6.0 (Graphpad Software, La Jolla, CA, USA).

## 3. Results

### 3.1. rmMCP-7 Protease Induces Endothelial Cell Invasion

**Figure 1:**
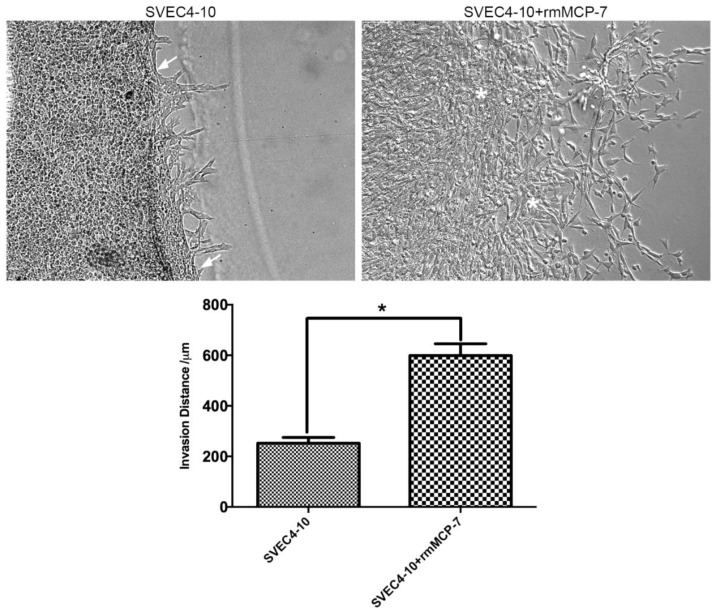
rmMCP-7 accelerates the invasion of endothelial cells into Geltrex^®^. In the absence of rmMCP-7, the boundary between the cells and the Geltrex^®^ can be seen (white arrows). In the cultures treated with rmMCP-7, the boundary between the cells and the Geltrex^®^ cannot be identified (asterisks). The distance covered by the cells was analyzed after 5 h of culture in the absence or presence of rmMCP-7. ImageJ was used to determine distance. The graph shows the mean invasion distance. Data from three independent experiments. * *p* < 0.05.

Since rmMCP-7 accelerates in vitro angiogenesis, it was of interest to determine whether the invasion of SVEC4-10 cells into Geltrex^®^ increased in the presence of rmMCP-7. When SVEC4-10 cells were incubated in the presence of rmMCP-7 using a Secure-Seal™ Hybridization Chamber Gasket attached to a Lab-Tek^®^ Chamber Slide™ filled with Geltrex^®^ an increase of about three-fold in the mean invasion distance of SVEC4-10 cells was observed compared to cells incubated in the absence of rmMCP-7. In the absence of rmMCP-7 the boundary between the gel and the endothelial cells can be identified. This does not occur in the presence of rmMCP-7, when the invasion process is accelerated (Figure 1).

### 3.2. rmMCP-7 Induces In Vivo Angiogenesis

**Figure 2:**
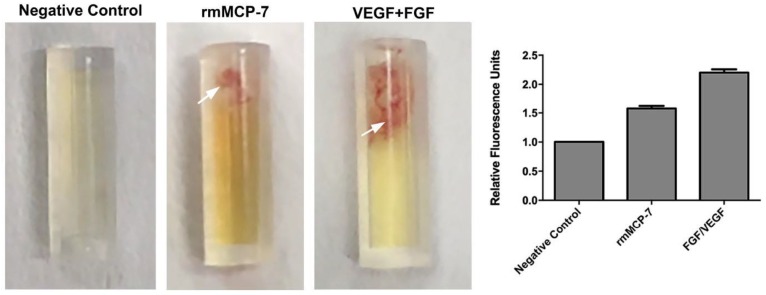
rmMCP-7 induces endothelial cell invasion into DIVAA™ inserts. Representative photographs demonstrate blood vessel growth (arrows) into the open end of the angioreactors only in the presence of rmMCP-7 or VEGF and FGF. DIVAA™ assay was performed in mice implanted with angioreactors containing Geltrex^®^ (negative control), or rmMCP-7 or VEGF and FGF (positive control). Graph shows the quantification of blood vessel growth, as measured by FITC-lectin fluorescence of the cell contents of each reactor.

The ability of rmMCP-7 to induce angiogenesis in vivo was assessed using the Directed In Vivo Angiogenesis Assay^TM^ (DIVAA^TM^). In the angioreactors containing only Geltrex^®^ (negative control), there was no blood vessel invasion into the angioreactor. Blood vessel invasion into the angioreactor was measured by lectin-FITC binding to the angioreactor contents. The fluorescence intensity of the contents of the angioreactor containing rmMCP-7 was 58% higher than the negative control, while the fluorescence intensity in the presence of VEFG+FGF (positive control) was 113% higher than the negative control. Thus, these findings demonstrate that rmMCP-7 is capable of inducing angiogenesis both in vivo (Figure 2) and in vitro.

### 3.3. rmMCP-7 Induces Changes in Integrin Subunit Levels during Angiogenesis

**Figure 3:**
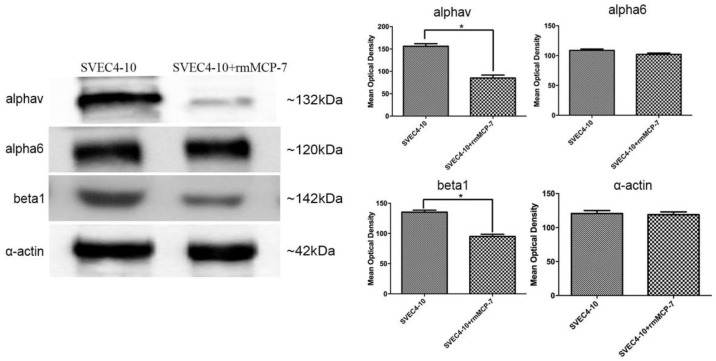
rmMCP-7 decreases the levels of αv and β1 integrin subunits during angiogenesis in vitro. α6 subunit levels remain unchanged. Integrin levels were analyzed by Western blot after tube formation assay. The graphs show the quantification of Western blot bands by densitometry. α-actin was used as a loading control. Data from three independent experiments. * *p* < 0.05

In order to understand the mechanism of action of rmMCP-7 during angiogenesis, the protein levels of integrin subunits αv, α6 and β1 were evaluated during angiogenesis in vitro, in the presence and absence of rmMCP-7. The results of the tube formation assay showed that there was a decrease in the protein levels of αv and β1 integrin subunits in the presence of rmMCP-7. The levels of the subunit α6 were unaltered (Figure 3). Therefore, during in vitro angiogenesis, the presence of rmMCP-7 can decrease integrin subunit levels.

### 3.4. rmMCP-7 Acts directly to Degrade Integrin Subunits

**Figure 4:**
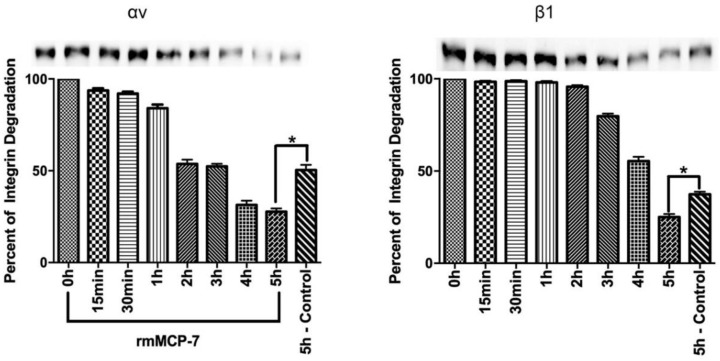
The αv and β1 integrin subunits were degraded by rmMCP-7. A basal degradation of the integrin subunits is observed in the absence of rmMCP-7 (control). The recombinant integrin was incubated for 5 h in the presence or absence (control) rmMCP-7. Integrin levels were analyzed by Western blot. The graphs show the quantification of Western blot bands by densitometry. Data from three independent experiments. * *p* < 0.05.

Since the levels of αv and β1 integrin subunits were decreased in the presence of rmMCP-7 during in vitro angiogenesis, it was of interest to determine if rmMCP-7 has the ability to directly degrade integrin subunits. Therefore, degradation assays were performed with recombinant αv and β1 integrin subunits. The results showed that the direct degradation of the αv and β1 subunits is increased about 35% after incubation for 5 h in the presence of rmMCP-7 (Figure 4), relative to the control (absence of rmMCP-7). These results indicate that the decreased levels of the αv and β1 subunits in the presence rmMCP-7 is due, at least in part, to direct degradation of the integrin subunits.

### 3.5. The Degradation of Integrin Subunits Occurs Indirectly via the Ubiquitin/Proteasome System

**Figure 5:**
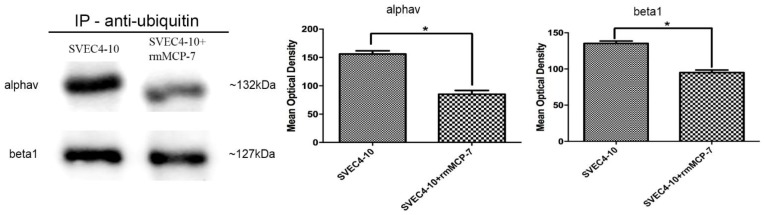
Both the αv and β1 subunits are ubiquitinated during angiogenesis in vitro. Note that the levels of integrin subunits are lower in the presence of rmMCP-7. Integrin levels were analyzed by Western blot after immunoprecipitation (IP) using anti-ubiquitin antibody after the tube-forming assay. Western blot bands were quantified by densitometry. Data from three independent experiments. * *p* < 0.05.

As degradation and recycling of integrins are required for angiogenesis and the ubiquitin/proteasome system controls the turnover of these proteins, experiments were performed to investigate if the αv and β1 integrin subunits were also ubiquitinated during in vitro angiogenesis. In either the presence or absence of rmMCP-7 both αv and β1 integrin subunits were ubiquitinated (Figure 5). Furthermore, in the presence of rmMCP-7 the protein levels of αv and β1 integrin subunits were lower during angiogenesis. These results indicate that the ubiquitin/proteasome system contributes to integrin subunit degradation. However, the most significant portion of integrin degradation in the presence of rmMCP-7 is due to the direct degradation by the protease.

### 3.6. rmMCP-7 Induces Angiogenesis Independent of the Ubiquitin/Proteasome System

**Figure 6:**
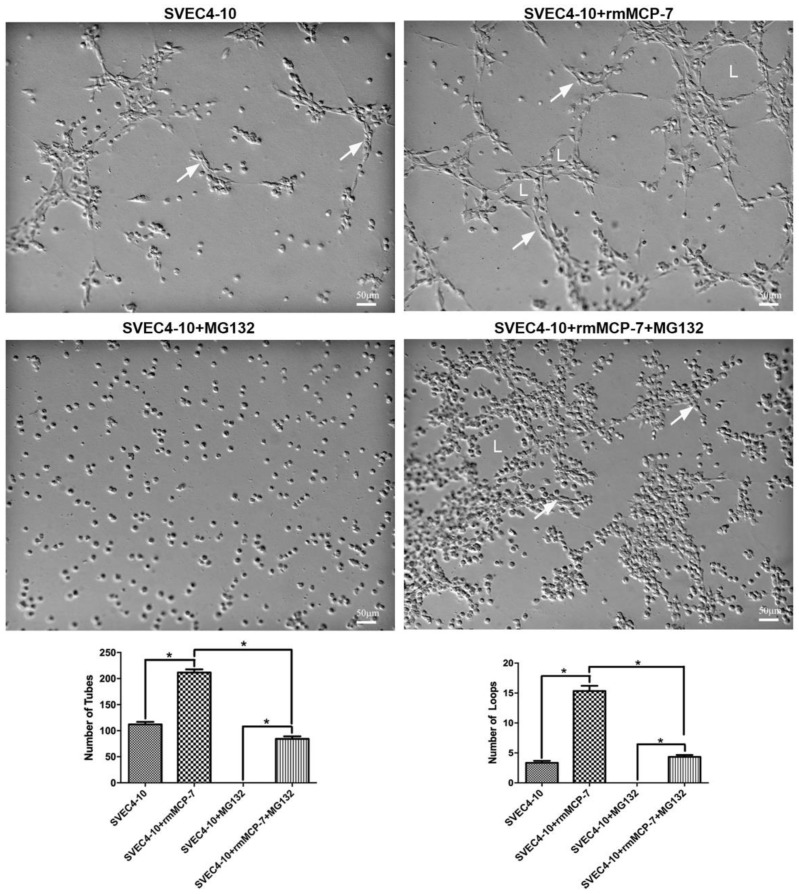
rmMCP-7 initiates tube formation even in the presence of the proteasome inhibitor MG132. When SVEC4-10 cells were cultured in the presence of MG132, in vitro angiogenesis was completely inhibited. However, when the cells were cultured with rmMCP-7 in the presence of MG132, in comparison to the cells incubated only with rmMCP-7 tube formation (arrows) is reduced. Furthermore, in the presence of rmMCP-7 and MG132, it is possible to observe a few cells spread on the substrate as well as some loops (L). The cells were incubated for 5 h. Quantification of tube and loop formation using WimTube Wimasis is shown in the graphs. * *p* < 0.05.

To confirm that the ubiquitin/proteasome system acts during angiogenesis, an in vitro tube-forming assay was performed in the presence of a proteasome inhibitor (MG132). Incubation of SVEC4-10 cells with MG132 resulted in a complete inhibition of in vitro angiogenesis (Figure 6). It was then of interest to determine if rmMCP-7 was capable of inducing in vitro angiogenesis in the absence of integrin turnover. In the presence of MG132, rmMCP-7 was still able to induce limited in vitro angiogenesis that included migration of endothelial cells, tube formation, and the formation of a few loops (Figure 6). Thus, the presence of rmMCP-7 is sufficient to induce endothelial cell migration and the initiation of tube formation in the presence of the inhibitor. Although the extensive spreading of endothelial cells and loop formation, characteristic of in vitro angiogenesis induced by rmMCP-7, was not observed (Figure 6).

## 4. Discussion

The current study demonstrates, for the first time, a functional role for rmMCP-7 in angiogenesis. The integrin subunits αv and β1 were directly degraded by rmMCP-7, thereby facilitating endothelial cell migration and invasion, processes that are essential for angiogenesis.

Previously, mast cell proteases which are stored in mast cell secretory granules have been shown to influence angiogenesis [29,33,66,67,68]. Various studies have demonstrated that tryptases act on a variety of substrates [55,58,69,70,71] and that tryptase activity is a key factor in angiogenesis where it is involved in degradation of the extracellular matrix [72,73], plasminogen activation [74], fibrinogen degradation [31,75,76,77], activation of latent collagenase and matrix metalloproteinases [78,79], and tryptase-induced endothelial barrier dysfunction [80,81]. Additionally, an increased number of mast cells has been demonstrated in angiogenesis where they are associated with vascular tumors, a number of hematological and solid lung tumors, as well as cutaneous cancer [82,83,84]. This possible relationship between mast cells and angiogenesis may also be mediated through several proangiogenic factors such as VEGF, FGF-2, PDGF, and interleukin-6 (IL-6) that are also stored in mast cell secretory granules [50,52,57,85,86,87].

Our results with rmMCP-7 corroborate previous studies that have demonstrated that integrins are associated with the invasion of endothelial cells into the extracellular matrix during angiogenesis [88,89]. Therefore, the integrin degradation observed in the presence of rmMCP-7 suggests that the protease may act directly or indirectly in the degradation of the integrin subunits inducing angiogenesis. Degradation of integrin subunits has been observed in various studies showing that recycling or degradation of different integrin subtypes is essential for integrin activation. Degradation increases the affinity of integrin receptors for their extracellular ligands [90,91].

In the present study, when the tube-forming assay was performed in the presence of a proteasome inhibitor (MG132), angiogenesis was inhibited. These results agree with previous studies showing that proteasome inhibitors can act on angiogenesis by inhibiting cell migration, tube formation and release of angiogenic mediators by endothelial cells [92,93,94]. In contrast, even in the presence of the proteasome inhibitor, incubation of endothelial cells with rmMCP-7 induced cell migration and tube formation as well as the beginning of loop formation. However, extensive endothelial cell spreading, and loop formation was not seen under these conditions. Therefore, the degradation of integrin subunits by rmMCP-7 is sufficient for the initiation of angiogenesis, but integrin turnover is required for the proper spreading of endothelial cells, as well as for complete loop formation. In the presence or absence of rmMCP-7 the αv and β1 integrin subunits were ubiquitinated. Recent data have demonstrated that proteasomal degradation of integrin receptors may also occur and may be regulated by the ubiquitin-proteasome pathway [91,95,96]. Lobert el al. [95] present evidence that a fraction of the α5β1 integrin is ubiquitinated and degraded together with fibronectin in a ligand-ESCRT-dependent manner, and that this is required for proper fibroblast migration. Hsia et al. [96] demonstrated that α5 integrin degradation is mediated by proteasomes and α5 integrin is associated with the ubiquitination pathway in the absence of fibronectin [97]. The ubiquitin/proteasome system controls the degradation and recycling of proteins involved in critical cellular functions, such as migration and adhesion [97,98,99].

The integrins link cells to the extracellular matrix and play an important role in sensing the environment and controlling cell shape and motility [100,101,102,103]. Several researchers have reported changes in the expression levels of integrins during angiogenesis, both in vitro and in vivo. However, the significance of variations in integrin activity, conformation and function during angiogenesis is still not completely understood. Walsh et al., 2009 [104] observed that differences in the levels of integrin subunit expression correlated with matrix invasion by human pancreatic cancer cells. Different experimental approaches, such as inhibition with antibodies, knock-in mutations and genetic deletions have led to conflicting theories on the role of integrins in angiogenesis [104]. Studies using antibodies against the αv integrin subunit suggested that this subunit promoted angiogenesis, while genetic deletion of the αv integrin subunit indicated that αv integrins are not required for angiogenesis [105,106,107,108,109].

Previous studies have shown an important role for α2β1 integrin in angiogenesis. San Antonio et al. [110] demonstrated that the small molecule inhibitor (SMI496) of α2β1 integrin interfered with angiogenesis by inhibiting sprouting from the axial vessels. They also showed that morpholino-mediated knockdown of α2 can alter angiogenesis, promoting the formation of nonfunctional vessels. Furthermore, when endothelial cells were plated on type I or type IV collagen (both α2β1 integrin-dependent ligands), fibronectin (an α2β1 integrin-independent ligand), or tissue culture plastic, and cell proliferation were evaluated, α2-null endothelial cells showed a four- to five-fold higher proliferative index when compared with their wild-type counterparts on all matrices [111]. Additionally, genetic ablation studies have demonstrated a critical role for β1 integrins in angiogenesis and vascular development [112,113]. These data indicate that β1 integrin is required for maintenance of endothelial cell shape, single layered morphology, and luminal patency of mid-sized arteries after formation of the major vessels [113].

Interestingly, the levels of the α6 integrin subunit were unaltered in the presence of rmMCP-7. The α6 integrin subunit forms part of the α6β1 and α6β4 integrin complexes, which can selectively bind to different ligands such as fibronectin, vitronectin, collagen, and laminin. They also are involved in different biological activities including embryogenesis, organogenesis, and invasion of carcinoma cells [114]. Primo et al. [115], studying the functional role of α6 integrin during angiogenesis by lentivirus-mediated gene silencing and blocking antibody, observed that the expression of α6 in endothelial cells during angiogenesis could be modified depending on the substrate.

Our results demonstrate, for the first time, a functional role for mMCP-7 in angiogenesis. This knowledge may be useful in developing future therapeutic targets. The scarcity of specific targets is the greatest challenge in controlling angiogenesis in diverse pathological processes.

## 5. Conclusions

Degradation of integrin subunits by mMCP-7 facilitates migration and invasion of endothelial cells, thereby promoting angiogenesis both in vitro and in vivo.
